# Natural genetic variation in *Drosophila melanogaster* reveals genes associated with *Coxiella burnetii* infection

**DOI:** 10.1093/genetics/iyab005

**Published:** 2021-01-23

**Authors:** Rosa M Guzman, Zachary P Howard, Ziying Liu, Ryan D Oliveira, Alisha T Massa, Anders Omsland, Stephen N White, Alan G Goodman

**Affiliations:** 1 School of Molecular Biosciences, College of Veterinary Medicine, Washington State University, Pullman, WA 99164, USA; 2 Department of Veterinary Microbiology and Pathology, College of Veterinary Medicine, Washington State University, Pullman, WA 99164, USA; 3 Paul G. Allen School for Global Animal Health, College of Veterinary Medicine, Washington State University, Pullman, WA 99164, USA; 4 USDA-ARS Animal Disease Research, Pullman, WA 99164, USA; 5 Center for Reproductive Biology, Washington State University, Pullman, WA 99164, USA

**Keywords:** genome-wide association study, bacteria, pathogenesis, immunity, host response

## Abstract

The gram-negative bacterium *Coxiella burnetii* is the causative agent of Query (Q) fever in humans and coxiellosis in livestock. Host genetics are associated with *C. burnetii* pathogenesis both in humans and animals; however, it remains unknown if specific genes are associated with severity of infection. We employed the *Drosophila* Genetics Reference Panel to perform a genome-wide association study to identify host genetic variants that affect host survival to *C. burnetii* infection. The genome-wide association study identified 64 unique variants (*P* < 10^−5^) associated with 25 candidate genes. We examined the role each candidate gene contributes to host survival during *C. burnetii* infection using flies carrying a null mutation or RNAi knockdown of each candidate. We validated 15 of the 25 candidate genes using at least one method. This is the first report establishing involvement of many of these genes or their homologs with *C. burnetii* susceptibility in any system. Among the validated genes, *FER* and *tara* play roles in the JAK/STAT, JNK, and decapentaplegic/TGF-β signaling pathways which are components of known innate immune responses to *C. burnetii* infection. *CG42673* and *DIP-ε* play roles in bacterial infection and synaptic signaling but have no previous association with *C. burnetii* pathogenesis. Furthermore, since the mammalian ortholog of *CG13404* (*PLGRKT*) is an important regulator of macrophage function, *CG13404* could play a role in host susceptibility to *C. burnetii* through hemocyte regulation. These insights provide a foundation for further investigation regarding the genetics of *C. burnetii* susceptibility across a wide variety of hosts.

## Introduction

*Coxiella burnetii* is the causative agent of Query (Q) fever, a zoonotic disease that poses a serious threat to both human and animal health (Maurin and Raoult 1999). Because of its morbidity, low infectious dose, and the environmental stability of *C. burnetii*, the US NIH and CDC classify it as a Category B priority pathogen (Madariaga *et al.* 2003). Humans primarily become infected from sheep, goats, and cattle through inhalation of contaminated aerosols (Marrie *et al.* 1996; McQuiston *et al.* 2002; Schimmer *et al.* 2008). Therefore, reducing bacterial load in the livestock is critical to preventing Q fever outbreaks. *C. burnetii* is endemic worldwide and sporadic outbreaks have recently been reported in the United States (Karakousis *et al.* 2006; Anderson *et al.* 2013; Kersh *et al.* 2013; Sondgeroth *et al.* 2013; Dahlgren *et al.* 2015). A recent large outbreak of Q Fever on a goat farm in the Netherlands cost 307 million Euros in public health management efforts and agricultural interventions (Schimmer *et al.* 2008; Roest *et al.* 2011a, 2011b; van Asseldonk *et al.* 2013). To date, no commercial Q fever vaccine is available for humans or animals in the United States, and antibiotic therapy is the only option for treating human infection. Culling infected or at-risk animals is often employed to control outbreaks (Roest *et al.* 2011b, 2012, 2013). Additionally, the lack of animal models with genetic malleability and the strict requirements in BSL3 animal facilities towork with Select Agent phase I virulent strains of *C. burnetii* make it difficult to study host–pathogen interactions *in vivo*.

Host genetics influence the development of *C. burnetii* infection in both humans and other animals (Ghigo et al. 2002; Leone et al. 2004; Raoult *et al.* 2005; Meghari *et al.* 2008; Delaby *et al.* 2012; De Lange *et al.* 2014). Experimental studies in human and mouse cells correlate defective monocyte/macrophage activation and migration with ineffective granuloma formation, and overexpression of interleukin (IL)-10 is present in patients with chronic Q fever (Meghari *et al.* 2008; Delaby *et al.* 2012; Bewley 2013; Mehraj *et al.* 2013; Ka *et al.* 2014). Two recent studies genotyped human populations and revealed that genetic variation in innate immune genes, such as those encoding pattern recognition receptors and *IFNG*, are associated with susceptibility to Q fever (Wielders *et al.* 2015; Ammerdorffer *et al.* 2016). Despite this importance, specific genetic variants associated with susceptibility to *C. burnetii* infection remain largely unknown. In addition, it is unknown how host genetic factors affect bacterial load and shedding in susceptible reservoir hosts, namely livestock.

Previous studies profiled mammalian host responses to *C. burnetii* infection. These studies show that the bacteria downregulate the host innate immune response during acute infection and determine that the resolution of Q fever is associated with the re-establishment of type I interferon (IFN) signaling (Ghigo et al. 2002; Faugaret *et al.* 2014; Gorvel *et al.* 2014). Directed studies in humans reveal that single-nucleotide polymorphisms (SNPs) in innate immune receptors and signaling genes such as *TLR1, STAT1, IFNG*, and *MYD88* are associated with acute or chronic Q fever (Schoffelen *et al.* 2015; Wielders *et al.* 2015). Since these studies used a targeted approach to examine SNPs in only a set of candidate genes, we aimed to undertake a global, genome-wide analysis to identify gene variants associated with *C. burnetii* infection using *Drosophila melanogaster* as the host model.

We recently demonstrated that adult *D. melanogaster* is susceptible to infection with the BSL2 NMII clone 4 strain of *C. burnetii* and that this strain replicates in flies (Bastos *et al.* 2017). Importantly, this previous work established *D. melanogaster* as a suitable model for studying host–pathogen interactions during *C. burnetii* infection despite the bacteria being a mammalian pathogen and not a natural pathogen of insects. Additionally, mechanisms of immunity differ between mammals and insects, most notably the lack of an adaptive immune response in insects. Furthermore, while many components of innate immunity are conserved between mammals and insects, such as TLR/Toll, NFκB/Relish, and JAK/STAT signaling (reviewed in Sheehan *et al.* 2018; Tafesh-Edwards and Eleftherianos 2020), insects lack an intact cGAS/STING signaling axis, as a cytosolic DNA sensor has yet to be identified in insects (Goto *et al.* 2018; Hua *et al*. 2018; Martin *et al.* 2018). Application of the *D. melanogaster/C. burnetii* model to the *Drosophila* genetics reference panel (DGRP), a fully sequenced, inbred panel of fly lines derived from a natural population, provides an efficient platform for genotype-to-phenotype associations via a genome-wide association study (GWAS) (Mackay *et al.* 2012; Huang et al. 2014). The DGRP has already been used to reveal genes associated with susceptibility/tolerance to other bacterial pathogens (Bou Sleiman *et al.* 2015; Howick and Lazzaro 2017; Wang *et al.* 2017).

In this study, we identified genetic variants in *D. melanogaster* that were associated with susceptibility or tolerance to *C. burnetii* infection. Specifically, from the different GWA analyses performed, we obtained a list of 64 unique variants associated with 25 candidate genes that were selected for validation studies. Our analyses reveal genes with sex-specific effects on susceptibility and tolerance that have functions associated with actin binding, transcriptional response, and regulation of G-proteins. We found that multiple genes within the decapentaplegic (DPP) pathway, which is homologous to the TGF-β pathway in mammals (Gelbart 1989) were associated with susceptibility to infection. Similarly, we identified Rho GEFs and TGF-β, which are associated with the development of the *Coxiella*-containing vacuole and pathogenesis in humans, respectively (Benoit *et al.* 2008a, 2008b; Aguilera *et al.* 2009; Pennings *et al.* 2015; Salinas *et al.* 2015; Weber *et al.* 2016). Importantly, all the candidate genes identified here have mammalian orthologs or highly conserved functions that allow for extrapolation to mammalian systems.

Of the 25 candidate genes we identified in the GWAS, 15 genes significantly affected host survival during *C. burnetii* infection in *D. melanogaster* null mutants, RNAi knockdown flies, or both. We also examined the effect of candidate SNPs using regulatory element analysis (modENCODE) and found that some were within transcription factor binding hot spots, putative enhancers, novel splicing, branch point variation, and codon usage variation that could explain how the variants affect host gene expression and ultimately infection outcome. Altogether, we utilize the DGRP to identify host genetic variants associated with sex-specific susceptibility or tolerance to *C. burnetii* infection. The human orthologs of the genes we identified may also play important roles in human immune cell regulation, highlighting the conserved nature of gene function between insect and mammalian models of *C. burnetii* infection.

## Materials and methods

### *Drosophila melanogaster* and *C. burnetii* stocks

Fly stocks were obtained from the Bloomington *Drosophila* Stock Center, the Vienna *Drosophila* Resource Center, Exelixis at Harvard Medical School, and the Kyoto Stock Center. Fly stocks were maintained at room temperature in standard meal agar fly food at 25**°**C and 65**°**C humidity. All fly strains are listed in Supplementary Table S1. *Coxiella burnetii* Nine Mile phase II (NMII) clone 4 RSA439 was propagated in Acidified Citrate Cysteine Medium 2 as previously described (Omsland *et al.* 2009). *Coxiella burnetii* stocks were quantified using quantitative real-time PCR to measure bacterial genome equivalent (GE), as previously described (Coleman *et al.* 2004).

### Fly infections and hazard ratio phenotype determination

Each DGRP line was separated into groups of 40 male and 40 female adult flies (2–7 days old) (Supplementary Tables S2 and S3) and injected with phosphate-buffered saline (PBS) or 10^5^ GEs of *C. burnetii* diluted in PBS to establish infection. We infected flies at a multiplicity of infection of 10^5^ GE *C. burnetii*/fly based on the previous study establishing *D. melanogaster* as a model for *C. burnetii* infection (Bastos *et al.* 2017). For injections, flies were anesthetized with CO_2_ and injected with 23 nL of bacteria or PBS using a pulled glass capillary and an automatic nanoliter injector (Drummond Scientific, Broomall, PA, USA), as previously described (Hiroyasu *et al.* 2018). Individual flies were injected at the ventrolateral surface of the fly thorax and placed into new vials. Male and female flies were housed in separate vials. After injection, survival was monitored daily for 30 days with the flies maintained at 25°C and 68% humidity. We used Prism v8.0 (GraphPad Software, Inc.) to determine hazard ratios and *P*-values [log-rank (Mantel-Cox) test] for survival curves for males and females from each DGRP line. All survival analyses take the full 30-day trial into account, and raw data for the DGRP lines are found in Supplementary File S1. Lines having less than 3% mortality in the mock-infected group were not included in downstream analyses (Chow *et al.* 2013).

### Genome-wide association using hazard ratios and candidate gene analyses

To determine phenotype-to-genotype association, hazard ratios were log_10_ transformed and submitted to the dgrp2 webtool (http://dgrp2.gnets.ncsu.edu/), which adjusts the phenotype for the effects of *Wolbachia* infection and major inversions (Mackay *et al*. 2012; Huang *et al*. 2014). Three separate analyses were run using male, female, and combined hazard ratios for the DGRP lines (Supplementary Tables S2-S4). *R* was used to create Quantile–quantile (Q–Q) plots from log-transformed hazard ratios and obtain *r*^2^ values and genomic inflation values (λ). For male and female analyses, 193 male and 195 female log-transformed hazard ratios were submitted (Supplementary Tables S2 and S3, respectively). SNPs and small indel variants with a *P*-value (mixed-effects model) below 10^−5^ were considered genome-wide suggestive candidate variants and further analyzed. For the combined analysis, both male and female hazard ratios were submitted for 191 DGRP lines. Candidate variants with *P*-values (mixed-effects model) less than 10^−5^ for the average trait or the difference (female–male) trait were selected for further study. Since the nominal genome-wide suggestive *P*-value of 10^−5^ has been employed in other GWA studies (Stranger *et al.* 2011; Chow *et al.* 2016; Fadista *et al.* 2016; Zhang *et al.* 2016; Gibson *et al.* 2019), this threshold justifies our choice for the purposes of this study and choosing candidate genes to evaluate further. The DGRP genome assembly (BDGP R5/dm3) was used to identify variants in candidate genes. Human orthologs of candidate genes were identified using the DRSC Integrative Ortholog Prediction Tool (DIOPT v8.0) reported in Flybase (Hu *et al.* 2011). The ortholog with the highest weighted score is reported in Table 1. Predicted functions for each candidate gene were gathered using Flybase (FB2019_02). Regulatory annotation summaries for each SNP and indel were compiled using Flybase (FB2019_02) and modENCODE utilizing variant coordinates converted to the BDGP R6/dm6 reference assembly. To define regulatory annotations, we reviewed publicly available data within modENCODE tracks, such as all noncoding features including transcription factor binding sites (TFBS), histone ChIP-seq data, chromatin domain segmentation, and small RNA-seq tracks. Relative male: female ratio gene expression results were calculated using RNA-seq data (Graveley *et al.* 2011) in Flybase. Sex-specific expression data of the exon nearest to the gene variant were averaged for 1- and 5-day-old flies.

**Table 1:** Candidate genes associated with top variants from GWA analyses

Candidate gene	Top variant (BDGP R5/dm3)	Type	*P*-value	Analysis	Male:female expression	Regulatory annotations	Putative gene function	**Human ortholog** ^*^
*CG34351*	2L_4702261_SNP	Intronic	7.5 × 10^-6^	Female	2.3	Poorly annotated Euchromatin transcriptionally silent (or intergenic)	Regulation of G-proteins	RGS7BP
*DIP-ε*	2L_6394872_SNP	Exonic; synonymous	2.9 × 10^-6^	Male	3.8	Euchromatin transcriptionally silent or dynamic	Interaction with Dprs	OPCML
*rk*	2L_13999491_SNP	Intronic	8.4 × 10^-6^	Difference	-	Transcriptionally silent	GPCR; buriscon receptor; melanization	LGR5
*shn*	2R_7099616_SNP	Exonic; synonymous	1.9 × 10^-7^	Female	1.4	TFBS hot spot	Zinc finger C2H2 transcription factor	HIVEP2
*GNBP-like3*	2R_16414194_SNP	Exonic; synonymous	6.1 × 10^-6^	Male	1.5	Euchromatin transcriptionally silent or dynamic	Beta 1,3-glucan recognition/binding	CRYBG1
*CG42741*	2R_18904195_SNP	Intronic	5.3 × 10^-6^	Difference	10	Transcriptionally silent	Zinc finger C2H2 transcription factor	KLF3
*trh*	3L_376337_SNP	Intronic	4.4 × 10^-7^	Difference	2.6	TFBS	bHLH-PAS transcription factor	NPAS1
*CG32264*	3L_3750617_SNP	Intronic	7.5 × 10^-6^	Average	2.6	Transcriptionally silent	Actin binding	PHACTR2
*RhoGEF64C*	3L_4738164_SNP	Intronic	7.4 × 10^-6^	Male	7	Euchromatin transcriptionally silent or dynamic	Rho guanyl-nucleotide exchange factor	ARHGEF3
*Pura*	3L_7623383_SNP	Intronic	2.2 × 10^-7^	Average	1.5	lncRNA	Rho guanyl-nucleotide exchange factor	PLEKHG4
*dally*	3L_8851042_SNP	Intronic	6.7 × 10^-6^	Average	1.9	Putative enhancer but not hot spot	Co-receptor for growth factors/morphogens	GPC5
*CG42673*	3L_9540740_SNP	Intronic	3.5 × 10^-6^	Difference	4.5	TFBS	Nitric-oxide synthase binding	NOS1AP
*dpr6*	3L_10044744_SNP	Intronic	3.8 × 10^-6^	Difference	0.035	Transcriptionally silent	Interaction with DIPs	CADM1
*AstC-R2*	3L_18481371_SNP	Exonic; synonymous	1.4 × 10^-6^	Difference	4.2	Between two TFBS	Allatostatin receptor	SSTR2
*ich*	3R_4787301_SNP	Intronic	9.3 × 10^-7^	Average	-	TFBS hot spot	Zinc finger C2H2 transcription factor	PRDM15
*FER*	3R_5218712_INS	Intronic	2.7 × 10^-6^	Female	3.8	Active enhancer	Protein tyrosine kinase activity	FER
*tara*	3R_12079260_SNP	Intronic	7.7 × 10^-6^	Female	1.1	Active enhancer, TFBS hot spot	Transcriptional co-regulator	SERTAD1
*CG31221*	3R_15278653_SNP	Intronic	6.4 × 10^-6^	Male	2.7	Near TFBS	LDL receptor	LRP1B
*loco*	3R_18456211_SNP	Antisense RNA	2.3 × 10^-6^	Average	9.6	Antisense RNA, enhancer, TFBS	Regulation of G-proteins	RGS12
*CG1544*	3R_27026419_SNP	Intronic	9.4 × 10^-6^	Difference	2.1	TFBS	Oxoglutarate dehydrogenase	DHTKD1
*CG34417*	X_6434578_SNP	Intergenic; 226 bp upstream	9.9 × 10^-6^	Average	—	TFBS hot spot, putative enhancer/promoter	Actin binding	SMTN
*CG12075*	X_8751630_SNP	Intronic	2.7 × 10^-6^	Difference	1	Silent chromatin state	Lipid signaling	-
*Smr*	X_12610055_SNP	Intronic	8.9 × 10^-6^	Average	0.25	TFBS hot spot	Chromatin binding; transcriptional regulation	NCOR1
*IP3K2*	X_13210675_SNP	Intronic	7.0 × 10^-6^	Average	2.4	Putative enhancer site	Calcium regulation; IP3 signaling	NET1
*CG13404*	X_14160126_SNP	Exonic; synonymous	6.1 × 10^-6^	Female	4.3	Active enhancer, TFBS hot spot	Plasminogen receptor (KT)	PLGKRT

* Human orthologs from DIOPT. Ortholog with highest weighted score reported.

### Validation of candidate genes

To empirically determine the effect of knockout or knockdown of the candidate gene on severity to infection, 40 adult flies from each null mutant and RNAi knockdown for each candidate gene were injected with PBS or *C. burnetii*, as stated previously. RNAi knockdown was performed using straight-winged progeny from crosses between the CyO-balanced *Act5C-GAL4* driver line and the corresponding dsRNA-containing RNAi lines (Supplementary Table S1). Sibling progeny flies carrying the CyO balancer were used as control flies. Genetic background strains for each null mutant strain were used as control flies. We conducted all survival experiments for each candidate gene twice independently and we used Prism v8.0 (GraphPad Software, Inc.) to determine hazard ratio and *P*-value [log-rank (Mantel-Cox) test] for each independent survival experiment to ensure that the two experiments were not significantly different from each other (data not shown). We then combined the results from both experiments to determine single hazard ratios and generate survival graphs (Ahlers *et al.* 2019; Dudzic *et al.* 2019). Raw survival data for validation experiments are found in Supplementary File S2. Significance levels of combined survival experiments for each genotype were binned into one of three categories: no significance (*P* > 0.01), low significance (0.01 > *P* > 0.0001), or high significance (*P* < 0.0001) (Supplementary Tables S6 and S7). We considered a gene to validate if the significance level changed between control and null mutant or RNAi knockdown genotypes for the sexes corresponding to GWA analysis type. For validated gene candidates in the average category, significance levels changed for both sexes, and validated gene candidates in the difference category changed for one sex but not the other.

### Splicing, branch point variation, and codon usage analysis

The Ensembl project (http://uswest.ensembl.org/index.html) and the Human Splicing Finder (http://www.umd.be/HSF/) were used to determine splicing and branch point variation from curated sequences to determine codon usage fraction based on frequency of amino acids per thousand.

### Data availability

Strains and stocks are available upon request. Genomic sequence for the DGRP is available at http://dgrp.gnets.ncsu.edu/. Supplementary material and all raw survival data (Supplementary Files S1 and S2) are available at FigShare: https://doi.org/10.25386/genetics.13490244. The authors affirm that all data necessary for confirming the conclusions of the article are present within the article, figures, and tables.

## Results

### Susceptibility to *C. burnetii* infection is dependent on host genetic background

**Figure 1:**
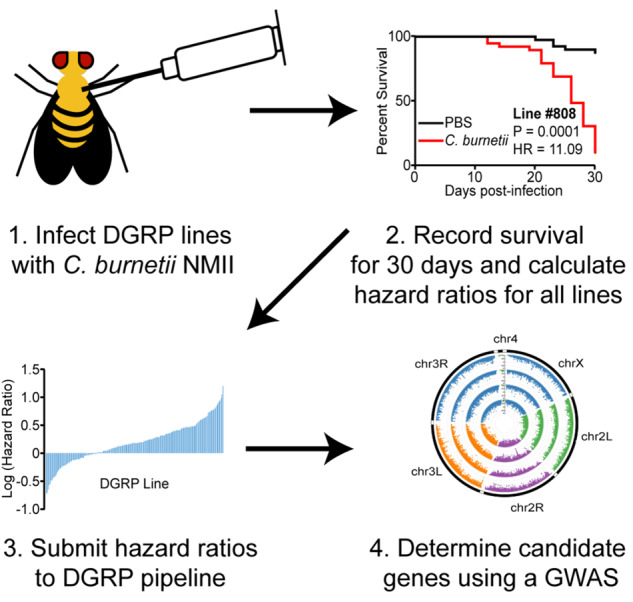
Experimental design schematic. Groups of 40 males and females per DGRP line were injected with PBS or *C. burnetii* at 10^5^ bacteria/fly and host survival monitored for 30 days to obtain hazard ratios. The hazard ratios of all DGRP lines were log-transformed and used as input for a GWAS.

Previously, we determined that flies deficient in the IMD signaling pathway genes, *PGRP-LC* and *Relish*, exhibit increased susceptibility to *C. burnetii* infection. We also determined that the gene *eiger* contributed to decreased tolerance to *C. burnetii* infection in flies, as *eiger* mutant flies were less susceptible to *C. burnetii* infection (Bastos *et al.* 2017). Therefore, we hypothesized that susceptibility to *C. burnetii* infection in *Drosophila* is associated with host genetics, and that the broad base genetic variation in the DGRP could identify other candidate genes that effect susceptibility to *C. burnetii* infection via a GWAS. To determine the susceptibility of each DGRP line to infection, adult males and females of each line were mock-infected or infected with *C. burnetii*. We then monitored survival and calculated hazard ratios that were used as input for the GWA analysis (Figure 1). In total, we calculated 193 and 195 hazard ratios for males and females, respectively. The survival curves reveal an approximately log-normal distribution of hazard ratios ranging from −0.719 to 1.643 for male flies (0.191–44.01, non-log-transformed) and −0.714 to 1.200 for female flies (0.1932–15.85, non-log-transformed) (Supplementary Tables S2 and S3, Figure S1A), which indicates that genetic polymorphisms in the DGRP lines affect susceptibility to *C. burnetii* infection. Interestingly, male flies are more susceptible than female flies overall to *C. burnetii* infection, with a mean hazard ratio of 1.90 for male flies and 1.56 for female flies (*P* = 0.0015) (Supplementary Figure S1A). Notably, we observe three distinct survival phenotypes for both male and female flies among all DGRP lines. These survival phenotypes are defined by the hazard ratio, which compares the mortality rate of *C. burnetii*-infected flies to mock-infected flies. Hazard ratio analysis has been used to examine flies’ mortality rate to West Nile virus compared to mock-infection and to *Pseudomonas entomophila* based on route of infection (Martins *et al.* 2013; Ahlers *et al.* 2019). In general, susceptible DGRP lines display increased mortality to *C. burnetii* infection compared to mock-infection and positive log_10_ hazard ratios. Tolerant DGRP lines show no change in survival between *C. burnetii* and mock-infection and have log_10_ hazard ratios close to zero. We also observe that certain DGRP lines exhibit decreased mortality compared to the mock-infected group, as noted by the negative log_10_ hazard ratio in Supplementary Tables S2 and S3. Negative hazard ratios following microbial infection are not uncommon. This means that the genetic background of the particular DGRP line results in increased survival compared to the mock/injury control (Martins *et al.* 2013; Ahlers *et al.* 2019). A study on dietary restriction also shows negative hazard ratios (McCracken *et al.* 2020).

### GWA analyses of DGRP hazard ratios reveal candidate gene variants

The DGRP facilitates rapid GWA analyses using a quantitative phenotype via submission of a data set to the online webtool (Mackay *et al*. 2012). To determine polymorphisms in the DGRP population that affect susceptibility to *C. burnetii*, we submitted hazard ratios for analysis. We log_10_-transformed the hazard ratios prior to submission for GWA analysis (Supplementary Figure S1A) to yield an approximately normal distribution (Shapiro–Wilk test, *P* > 0.1) due to GWA analyses relying on parametric tests (Chow *et al.* 2013). We determined that hazard ratios are significantly positively correlated between male and female flies (*P* = 4.99 × 10^−7^), but with an *r*^2^ value of 0.121, which indicates a weak correlation and potential sex-dependent genotypes (Supplementary Figure S1B). Thus, we submitted hazard ratios as separate files for male and female analyses, and a single, combined file in order to identify polymorphisms that may be sex-dependent and to increase power for polymorphisms that are sex-independent. We termed the sex-independent analysis the average analysis, which results in top candidate variants that affect both sexes while the sex-dependent analysis which we termed difference analysis, results in top candidates that affect one sex but not the other. In total, we submitted 193, 195, and 191 hazard ratios for males, females, and average and difference, respectively (Supplementary Tables S2–S4).

**Figure 2:**
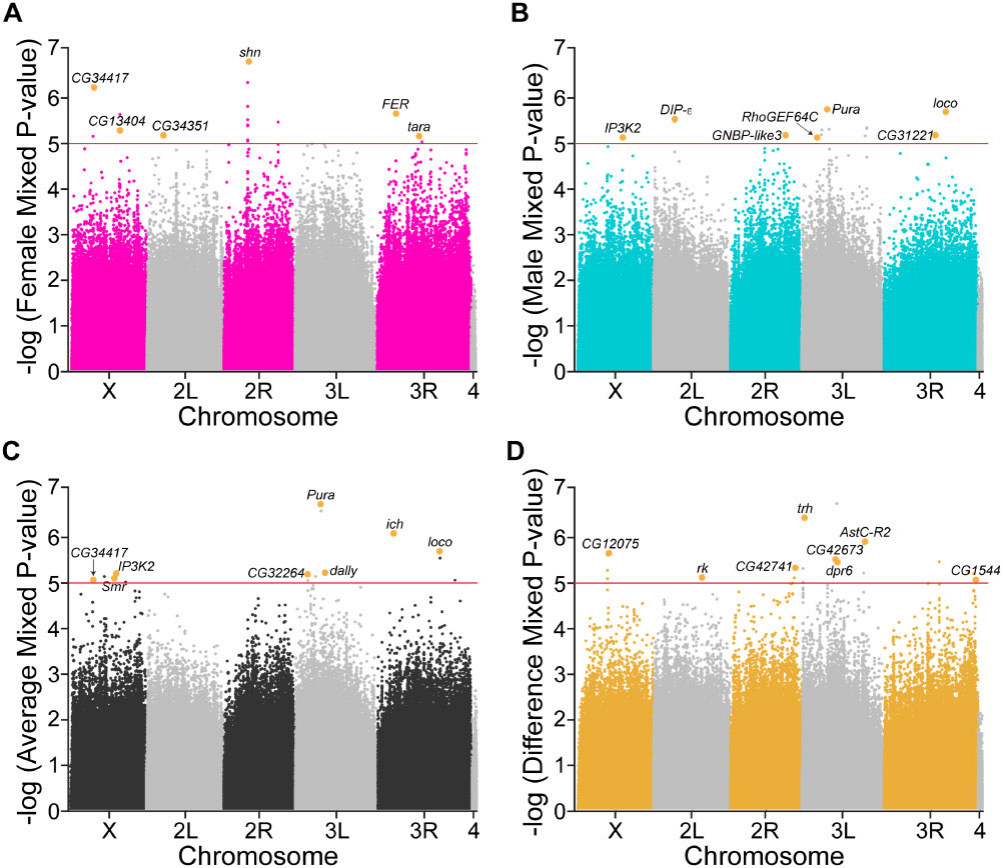
Genome-wide association analyses with hazard ratios reveal candidate genes. Manhattan plots for (A) male, (B) female, (C) average, and (D) difference GWA analyses using mixed-effect *P*-values for all four traits from dgrp2 webtool. Highlighted gene variants with *P*-values below 10^−5^ are labeled with associated candidate genes.

We tested a total of 1,893,791 polymorphisms in the male analysis, 1,897,049 polymorphisms in the female analysis, and 1,889,141 polymorphisms in the combined analysis. These analyses were not sufficiently powered to detect polymorphisms at a Bonferroni-corrected *P*-value of 2.64 × 10^−8^. Therefore, we employed a genome-wide suggestive *P*-value threshold of 10^−5^ which has been used for studies employing the DGRP (He *et al.* 2014; Chow *et al.* 2016; Kelsey and Clark 2017; Schmidt *et al.* 2017; Lavoy *et al.* 2018; Mackay and Huang 2018; Palu *et al.* 2020; Talsness *et al.* 2020). Using this *P*-value, we obtained a total of 69 associated polymorphisms from the GWA analyses, which included five duplicate variants (Figure 2 and Supplementary Table S5). Q–Q plots revealed no significant inflation due to dataset distribution, lambda values ranged from 0.993 (females) to 1.002 (difference), and *P*-values derived from these analyses appear to be reduced overall based on the lines from the Q–Q plots and lambda values below 1 (Supplementary Figure S2, A–D). Another interpretation of the Q–Q plots and GWAS significance values is that there is random association of our genome-wide suggestive variants. However, previous work has shown that weak signals in DGRP studies produce meaningful results. For example, candidate gene association and phenotypic correlation can be preserved among GWAS from different labs and using different populations of inbred fly lines (Everman *et al.* 2019; Pitchers *et al.* 2019). Furthermore, weak DGRP signals have been used to identify genes that genuinely regulate the phenotypic output of the DGRP studies (Chow *et al.* 2016; Palu and Chow 2018; Ahlers *et al.* 2019; Palu *et al.* 2020). Nevertheless, we calculated FDR corrections that would result in an equivalent *P*-value cutoff of *P* < 10^−5^ for each of our GWA analyses (Benjamini and Hochberg 1995; Pinheiro *et al.* 2020). For the female GWAS, the FDR correction is 0.88; for the male, it is 0.94; for the average, it is 0.99; for the difference, it is 0.78. As described in Everman *et al.* (2019), an FDR equivalent for a *P* < 10^−5^ cutoff ranged from 0.49 to 0.82 for previously published studies (Mackay *et al*. 2012; Everman and Morgan 2018). One reason our FDR corrections are higher is because our study uses additional DGRP lines than those cited above, which results in a greater number of tests (N) and additional variants with MAFs > 0.05. Nevertheless, the very lenient *P*-value cutoff we used gives us the opportunity to test more candidate genes in our validation experiments to rule out false positives that may have been selected using our lenient cutoff. While the DGRP may be underpowered, the ease with which one can perform empirical validation using *Drosophila* genetics makes the model as a whole very useful. Importantly, our goal is not to claim the associations reported here as definitive markers for host susceptibility to *C. burnetii* infection but to broadly identify candidate genes for future mechanistic studies in the context of *C. burnetii* or other pathogenic infections.

Of the 64 unique polymorphisms identified from the GWA, 14 variants are intergenic (21.9%), three of which are within 200 base pairs upstream of nearest gene; 39 are within introns (60.9%); eight are within exons (12.5%); one is within the 5ʹ UTR (1.6%); and two are in antisense-coding RNA within exon/introns (3.1%) (Supplementary Table S5). Of the eight SNPs within exons, six are silent and two are missense mutations. The 64 unique variants correspond to 31 unique genes that we narrowed to 25 candidate genes for validation experiments. Candidate genes were chosen based on stocks available and the location and type of gene disruption used in the available stocks. Due to these limitations, we did not perform validation experiments for *CG42455*, *side*, *MtnB*, *Or92a*, *Cpr100A*, or *CG32694*. We also report the relative male: female ratio gene expression data of each candidate gene using information available on Flybase (FB2019_02) (Table 1). We used the DGRP genome assembly (BDGP R5/dm3) to gather putative functions and regulatory annotations for each gene using Flybase and modENCODE and found that 12 are in TFBS (48%); nine are within regions predicted to be transcriptionally silent (36%); one is within a long noncoding RNA (4%); and three are in enhancers only (12%) (Table 1). Lastly, we report the human ortholog with the highest weighted score from the DRSC Integrative Ortholog Prediction Tool (DIOPT) on Flybase.

### Validation of candidate genes

**Figure 3:**
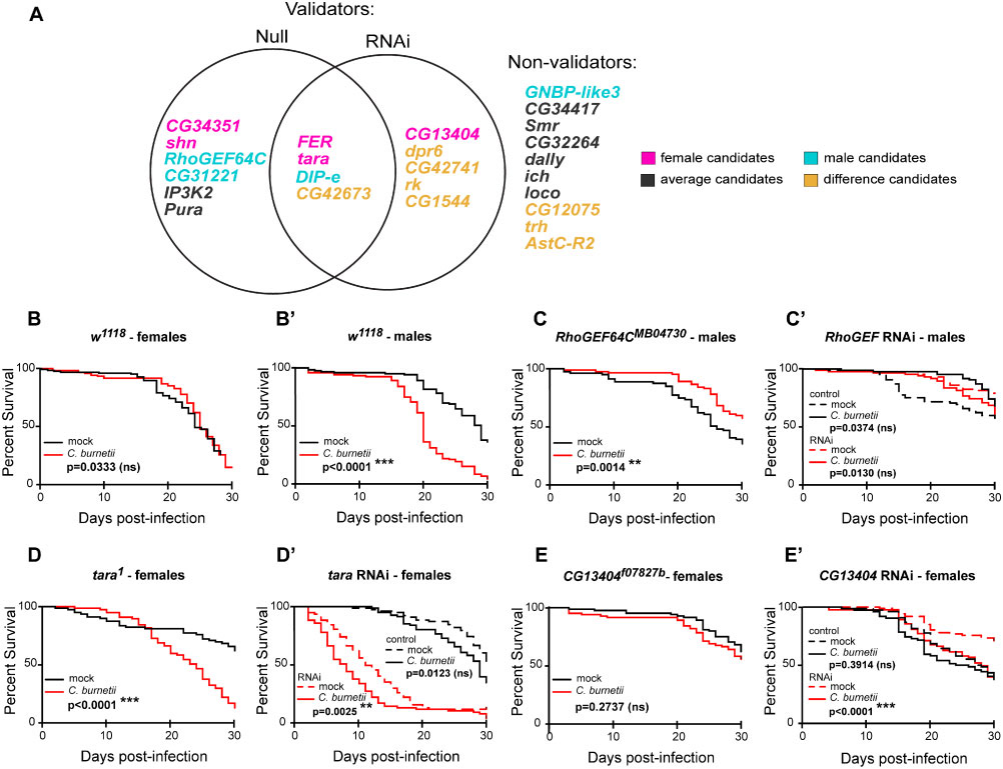
Fifteen GWAS candidate genes are validated using survival rate as a metric. (A) Venn diagram summarizes the genes that validate in null mutant flies, RNAi knockdown flies, or both. A gene is validated if the *P*-value for its survival curve (mock *vs* infected) changes between the control and experimental line. *P*-value thresholds were not significant (n.s., *P* > 0.01), *P* < 0.01, and *P* < 0.0001. Colors indicate the type of GWAS analysis from which the gene came. (B–Bʹ) Survival curves of control *w^1118^* (B) females and (Bʹ) males following mock or *C. burnetii* infection. (C–E) Survival curves of *RhoGEF64C^KG02832^* (C) or control and *RhoGEF64C* RNAi males (Cʹ), *tara^1^* (D) or control and *tara* RNAi females (Dʹ), or *CG13404^f07827a^* (E), or *CG13404* RNAi females (Eʹ) following mock or *C. burnetii* infection. Each survival curve represents two independent experiments of at least 40 flies that were combined for a final survival curve, Statistical significance (Log-rank test) from the mock-infected group is indicated.

We next tested the 25 candidate genes from the different GWA analyses (Table 1) by infecting and monitoring survival during *C. burnetii* infection for 30 days in flies carrying a null mutation in the candidate gene or knocked down for the candidate gene by RNAi. We defined validation of candidate genes when the null mutant or RNAi knockdown line that has a different threshold of survival *P*-value significance than its genetic control, as described in the *Materials and methods* section and Supplementary Tables S6 and S7. Of the 25 candidate genes, 6 validated in null mutants only (24%), five in RNAi knockdown only (20%), 4 in both null mutants and RNAi (16%), and 10 did not validate with either method (40%) (Figure 3A). Survival of *w^1118^* males and females (Figure 3, B–Bʹ) during *C. burnetii* infection was used as the genetic control for several null mutants, including *RhoGEF64C^MB04730^* (Figure 3C), *tara^1^* (Figure 3D), and *CG13404^f07827b^* (Figure 3E). We selected these candidate genes to represent how we determined validation based on *P*-value and survival trend for validating genes from different categories, i.e. null-only, RNAi-only, or both. *w^1118^* females (Figure 3B) are not susceptible to *C. burnetii* infection (*P* = 0.0333) but *w^1118^* males (Figure 3Bʹ) are highly susceptible (*P* < 0.0001) which corroborates our previous work (Bastos *et al.*, 2017). We selected the candidate gene *RhoGEF64C^MB04730^* from male-only GWA (Figure 3, C–Cʹ) and we observe that survival in null mutants (Figure 3C) is overall tolerant (*P* = 0.0014) compared to *w^1118^* males (Figure 3Bʹ). In contrast, there is no significant change in survival between control and RNAi-knockdown flies (Figure 3Cʹ) (control, *P* = 0.0374; RNAi, *P* = 0.0130). Thus, *RhoGEF64C^MB04730^* males validated only in null mutants. The candidate gene *tara* was selected from the female-only GWA and we observe that in null mutants (Figure 3D) and RNAi knockdown flies (Figure 3Dʹ), the absence of the gene results in decreased survival compared to control genotypes. Specifically, *tara*^1^ females are susceptible to infection (*P* < 0.0001) compared to *w^1118^* females (Figure 3B) and *tara* RNAi females (Figure 3Dʹ) are also susceptible (*P* = 0.0025) compared to control (*P* = 0.0123). Thus, *tara* validated for females in both null mutants and RNAi knockdown flies. We selected the candidate gene *CG13404 ^f07827b^* from female-only GWA and we observe that null mutants (Figure 3E) are not susceptible to infection (*P* = 0.2737) like *w^1118^* females (Figure 3B). In contrast, *CG13404* RNAi females (Figure 3Eʹ) are susceptible to infection (*P* < 0.0001) while control genotype females are not (*P* = 0.3914). Thus, *CG13404* validated only in RNAi knockdown flies. Finally, to determine the relative effects on survival in males and females for all candidate genes, we calculated the relative hazard ratio of the null mutant (Supplementary Figure S3A) or RNAi knockdown (Supplementary Figure S3B) flies as compared to their respective genetic controls. Relative hazard ratios greater than one mean that the loss of that gene reduces survival to *C. burnetii* infection while relative hazard ratios less than one mean that that the loss of that gene improves survival to *C. burnetii* infection.

### ENCODE analysis of validated genes

Splicing and branching of precursor mRNA and abundance of tRNA codons are known to affect gene expression (Královičová *et al.* 2004; Wang and Burge 2008; Sauna and Kimchi-Sarfaty 2011; Will and Luhrmann 2011; Singh and Cooper 2012; Komar 2016; Zhou *et al.* 2016; Jeacock *et al.* 2018). Therefore, we used data available from the ENCODE project to determine regulatory annotations for the variants in genes that validated in host survival experiments. Table 2 summarizes the splicing and branch point analysis in terms of percent variation from wild type and codon usage as a fraction of frequency of amino acid (SNP) per thousand over frequency of amino acid (wild type) per thousand. Several SNPs varied at the predicted mRNA splicing sites, branch points, or codon usage compared to wild-type sequences such as the variants affecting the validated genes *CG34351*, *DIP-ε*, *Pura*, *tara*, *FER*, and *IP3K2*. The insertion (3R_5218712) within *FER* results in a −92.69% difference from wild-type splicing and has no variation in branch point splicing from wild type. This change in splicing for the *FER* indel indicates the site is broken but a 3 base pair insertion offsets the destruction. Similarly, the SNP (3R_12079260) within *tara* differs 64.82% from wild-type splicing which also indicates a new splice site creation with no destruction. The frequency of the wild-type *DIP-ε* codon is 3.72-times higher than that of the *DIP-ε* SNP (2L_6394872), which suggests that decreased abundance of tRNA codon availability for the transcript variant may affect its translation and thus its function during *C. burnetii* infection. Changes in codon usage fraction for *shn* and *CG13404* may affect these gene variants albeit to a lesser extent.

**Table 2:** Splice, branch point, and codon usage analysis of validating genes

Candidate gene	Top SNP (BDGP R5/dm3)	Splice variation from wild type	Branch point variation from wild type	Codon change	**Codon usage fraction (wild type/SNP)** ^*^
*CG34351*	2L_4702261_SNP	No difference	−34.14%	—	—
*DIP-ε*	2L_6394872_SNP	N/A	N/A	Ctg/Ttg (silent)	3.72
*shn*	2R_7099616_SNP	N/A	N/A	ttA/ttG (silent)	1.1
*Pura*	3L_7623460_SNP	7.74%	No difference	—	—
*tara*	3R_12079260_SNP	64.82%	N/A	—	—
*FER*	3R_5218712_INS	−92.69%	No difference	—	—
*IP3K2*	X_13210675_SNP	−17.01%	No difference	—	—
*CG13404*	X_14160126_SNP	N/A	N/A	ctC/ctT (silent)	0.86

* Frequency of amino acid per thousand.

## Discussion

In this study, we describe the application of an unbiased GWAS using the DGRP to reveal variants in genes associated with *C. burnetii* infection. We show that 15 genes conferred a significant difference in host survival during *C. burnetii* infection in null mutants, RNAi knockdown, or both gene disruption methods. We also compiled regulatory annotations of the variants in validating genes and show that certain gene variants affect splicing and codon usage, which may in turn downregulate gene expression. These data support the use of previously generated null mutant or RNAi knockdown gene disruption fly stocks to validate our candidate genes. While *C. burnetii* is not a natural *D. melanogaster* pathogen, there is evidence that it is an endosymbiont of tick arthropods and may have co-evolved in these animals (Duron *et al.* 2015). Previous studies show that few genome-wide significant variants are identified when using a non-natural versus natural *D. melanogaster* pathogen (Magwire *et al.* 2012), or when the pathogen has low prevalence in *D. melanogaster* (Chapman *et al.* 2020). Similarly, our GWA analyses did not identify any variants that met the expected Bonferonni-corrected *P*-value significance level. Instead, this study provides a platform to examine potential host factors that regulate *C. burnetii* infection given the limited genetic tools available for the bacteria. It is currently not possible to perform a genetic screen in mammals using the BSL2 strain of *C. burnetii* due to the inability of the strain to infect wild-type animals. Furthermore, a genetic screen using a Select Agent strain would encounter its own logistical roadblocks. Using our innovative approach, we uncover genes and processes that are relevant to bacterial infections in general. Additionally, directed studies of these genes could be performed using a Select Agent strain of *C. burnetii.* For these reasons, we do not take the validating genes in this study to be absolute but rather a first step toward understanding their cross-species role in the host response to infection.

Previous studies have shown that *C. burnetii* infection differentially affects male and female mice (Leone et al. 2004; Textoris *et al.* 2010), and our results corroborate these studies. First, male and female hazard ratios differ significantly among the DGRP lines in the initial screen (Supplementary Figure S1A). We interpret the difference in hazard ratios between sexes as a conserved phenotype with mammalian organisms. Secondly, expression of the candidate genes in adult flies differs between sexes (Table 1). For example, the expression of *RhoGEF64C* and *DIP-ε* are 7.0 and 3.8-times more expressed in males than females, which is a potential reason why they validated in the male-only category, but not a definite cause. Validating genes from the difference category have higher differential sex expression, such as *CG42741* and *CG42673*, which are 10 and 4.5-times more expressed in males than females, respectively. In contrast, validating genes in average category have closer relative expression, such as *Pura* and *IP3K2*, which are only 1.5 and 2.4-times more expressed in males than females, respectively. These results suggest that gene expression may drive sex-specific differences in host survival to *C. burnetii* infection. Lastly, genes we identified and validated as sex-specific, such as *CG13404* and *FER*, have known functions in immunity, as discussed below.

Host survival in *CG13404* RNAi-knockdown female flies indicated they were significantly more susceptible to infection compared to control genotype. The human ortholog of *CG13404* is the plasminogen receptor gene *PLGRKT*, which is important for macrophage polarization and efferocytosis, two key components of inflammation regulation (Vago *et al.* 2019). The absence of Plg-R_KT_ causes defective plasminogen binding and inflammatory macrophage migration in both male and female mice pups, but only female *PLGRKT^−/−^* pups die 2 days after birth (Miles *et al.* 2017). Our results corroborate this study because *CG13404* was a top candidate from the female-only GWA. We hypothesize the role of Plg-R_KT_ in macrophage regulation connects CG13404 to immunity. In *D. melanogaster* immunity, hemocytes are the professional phagocytic cells. They are present in flies during both larval and adult stages, and they recognize, engulf, and destroy dying host cells and pathogens (Hoffmann 2003; Yano *et al.* 2008; Regan *et al.* 2013). Hemocytes are critical for innate immune signaling by mediating the secretion of antimicrobial peptides (AMPs) in response to pathogens through the Toll, JAK/STAT, and Immune deficiency (Imd) pathways (Hoffmann 2003; Lemaitre and Hoffmann 2007). Recent studies in our lab show that hemocytes support *C. burnetii* replication and induce Imd-specific AMPs (Bastos *et al.* 2017; Hiroyasu *et al.* 2018). However, our screen did not identify any genes in the classical Imd or Toll pathway. Nevertheless, the involvement of CG13404, the fly ortholog of Plg-R_KT_, during *C. burnetii* infection may exemplify conserved, sex-specific differences in mammalian macrophage and fly hemocyte regulation.

*FER* expression leads to activation of the DPP-mediated pathway, which has recently been shown to improve survival of *Klebsiella pneumoniae* through *STAT3* when overexpressed (Murray 2006; Dolgachev *et al.* 2018; Li *et al.* 2019). *Coxiella burnetii* induces expression of *STAT3* and *IL-10* during murine infection (Murray 2006; Textoris *et al.* 2010; Millar *et al.* 2015). Textoris *et al.* show that male mice have increased gene expression of *STAT3* and *IL-10* during infection which may account for the higher susceptibility of Q fever observed in men. Our study corroborates this study because *FER* was a top candidate in the female-only GWAS. We hypothesize that the absence of *FER* in females disrupts the immune response required to control infection and leads to significantly decreased host survival.

In addition to *FER*, the other three genes that validated in both gene disruption methods reveal new connections between immunity and *C. burnetii* infection. *Tara* encodes a transcriptional co-regulator that interacts with chromatin remodeling complexes, cell cycle proteins, the JNK signaling pathway, and plays a role in ataxin-1-induced degeneration (Fernandez-Funez *et al.* 2000; Calgaro *et al.* 2002; Branco *et al.* 2008; Afonso *et al.* 2015). The human ortholog of *tara* is *SERTAD1*, which is also a transcriptional co-regulator (Biswas *et al.* 2010; Savitz *et al.* 2013). Interestingly, induction of *SERTAD1* is expressed independently of IFN during Nipah virus infection (Glennon *et al.* 2015). IFN induction is tissue-dependent during *C. burnetii* infection (Hedges *et al.* 2016); therefore, it is plausible that *tara* is targeted by the bacteria during infection. *DIP-ε* encodes a protein belonging to the immunoglobulin superfamily of defective proboscis extension response (Dpr) and Dpr-interacting proteins (DIP), which form a complex network of cell surface receptors in synaptic specificity. The human ortholog of *DIP-ε* is *OPCML*, an immunoglobulin protein best characterized as a tumor suppressor (Cui *et al.* 2008; Birtley *et al.* 2019), and there is currently no reported role for either gene during bacterial infections. *CG42673* remains uncharacterized, but another DGRP GWAS reports that loss of function of *CG42673* in blood cells significantly impairs the cellular immune response to *Staphylococcus aureus* (Nazario-Toole 2016). Interestingly, this study also shows that *dpr10* significantly affects *S. aureus* phagosome maturation while our own top candidate, *dpr6* validated by RNAi knockdown. It is possible that *CG42673* functions as an enhancer like its human ortholog *NOS1AP* (Grossmann *et al.* 2015; Hein *et al.* 2015) and regulates *C. burnetii* infection.

While some gene candidates validated as expected, other candidates validated as per our statistical tests, but in opposite directions. For example, we observed differences between the DGRP predictive effect of top candidates and validating genes. The GWA output predicts that the *RhoGEF64C* SNP (3L_4738164) has increased host susceptibility to infection (effect = −0.1709, Supplementary Table S5). However, survival of *RhoGEF64C^MB04730^* null mutant males was significantly improved compared to control genotype (Figure 3C) during *C. burnetii* infection. The opposite survival trend in the null mutant flies is likely if the SNP is a gain-of-function mutation, which is difficult to test but worth pursuing in further studies. Similarly, the SNP (X_14160126) in *CG13404* (effect = 0.1213) predicts decreased host susceptibility to infection but RNAi knockdown females were significantly more susceptible compared to the control genotype (Figure 3Eʹ). One explanation for these opposing results is the possibility of gene product threshold effects, and overall susceptible or tolerant phenotypes during infection must be tested at the host level with subsequent functional experiments. Furthermore, the effect of the gene variants cannot be inferred from GWA alone, and knockout or knockdown of the associated gene may yield a different phenotype than that which was predicted for the variant in question. The use of null mutants or gene knockdown by RNAi is common practice to validate DGRP candidate gene variants (Howick and Lazzaro 2017; Palu *et al.* 2019, 2020), but the most precise way to test the effect that a gene variant has is to use gene-editing technology to knock-in the specific gene variant (Yoo *et al.* 2020). While we did not test the effect of each individual allele variant on host survival during *C. burnetii* infection, we conclude that the presence or absence of the genes in which the variants lie affects the outcome of infection.

In conclusion, this study builds on our previously developed framework utilizing *D. melanogaster* as an animal model to dissect the innate immune response to *C. burnetii* infection (Bastos *et al.* 2017; Hiroyasu *et al.* 2018). We observe that *C. burnetii* infection significantly depends on host genetic background of the fly. In contrast, genetic studies in relevant natural hosts such as ticks, livestock, and humans are severely limited. Thus, we propose that the validating genes in this study can be used to test new hypotheses regarding host responses, taking into consideration the genes’ function in flies, their regulatory annotations, and their orthologs’ function in humans or other animals. These studies may reveal novel mechanisms of transmission among different host species or help identify at-risk human and livestock populations through genotyping efforts.

## References

[iyab005-B1] Afonso DJS , Liu D, Machado DR, Pan H, Jepson JEC, et al 2015. TARANIS functions with cyclin A and Cdk1 in a novel arousal center to control sleep in Drosophila. Curr Biol. 25:1717–1726. 10.1016/j.cub.2015.05.037. 26096977 PMC4559600

[iyab005-B2] Aguilera M , Salinas R, Rosales E, Carminati S, Colombo MI, et al 2009. Actin dynamics and Rho GTPases regulate the size and formation of parasitophorous vacuoles containing *Coxiella burnetii*. Infect Immun. 77:4609–4620. 10.1128/IAI.00301-09. 19635823 PMC2747940

[iyab005-B3] Ahlers LRH , Trammell CE, Carrell GF, Mackinnon S, Torrevillas BK, et al 2019. Insulin potentiates JAK/STAT signaling to broadly inhibit flavivirus replication in insect vectors. Cell Rep. 29:1946–1960.e5. 10.1016/j.celrep.2019.10.029. 31722209 PMC6871768

[iyab005-B4] Ammerdorffer A , Stappers MH, Oosting M, Schoffelen T, Hagenaars JC, et al 2016. Genetic variation in TLR10 is not associated with chronic Q fever, despite the inhibitory effect of TLR10 on Coxiella burnetii-induced cytokines in vitro. Cytokine. 77:196–202. 10.1016/j.cyto.2015.09.005. 26364993

[iyab005-B5] Anderson A , Bijlmer H, Fournier PE, Graves S, Hartzell J, et al 2013. Diagnosis and management of Q fever–United States, 2013: recommendations from CDC and the Q Fever Working Group. MMWR Recomm Rep. 62:1–30. 23535757

[iyab005-B6] Bastos RG , Howard ZP, Hiroyasu A, Goodman AG. 2017. Host and bacterial factors control susceptibility of *Drosophila melanogaster* to *Coxiella burnetii* infection. Infect Immun. 85:e00218-17 doi:10.1128/IAI.00218-17. 10.1128/IAI.00218-17 PMC5478956 28438980

[iyab005-B7] Benjamini Y , Hochberg Y. 1995. Controlling the false discovery rate: a practical and powerful approach to multiple testing. J R Stat Soc B (Methodol). 57:289–300. 10.1111/j.2517-6161.1995.tb02031.x.

[iyab005-B8] Benoit M , Barbarat B, Bernard A, Olive D, Mege J-L. 2008a. *Coxiella burnetii*, the agent of Q fever, stimulates an atypical M2 activation program in human macrophages. Eur J Immunol. 38:1065–1070. 10.1002/eji.200738067. 18350541

[iyab005-B9] Benoit M , Ghigo E, Capo C, Raoult D, Mege J-L. 2008b. The uptake of apoptotic cells drives *Coxiella burnetii* replication and macrophage polarization: a model for Q fever endocarditis. PLoS Pathog. 4:e1000066. 10.1371/journal.ppat.1000066. 18483547 PMC2361190

[iyab005-B10] Bewley KR. 2013. Animal models of Q fever (*Coxiella burnetii*). Comp Med. 63:469–476. 24326221 PMC3866982

[iyab005-B11] Birtley JR , Alomary M, Zanini E, Antony J, Maben Z, et al 2019. Inactivating mutations and X-ray crystal structure of the tumor suppressor OPCML reveal cancer-associated functions. Nat Commun. 10:3134. 10.1038/s41467-019-10966-8. 31316070 PMC6637204

[iyab005-B12] Biswas SC , Zhang Y, Iyirhiaro G, Willett RT, Rodriguez Gonzalez Y, et al 2010. Sertad1 plays an essential role in developmental and pathological neuron death. J Neurosci. 30:3973–3982. 10.1523/JNEUROSCI.6421-09.2010. 20237268 PMC2861041

[iyab005-B13] Bou Sleiman MS , Osman D, Massouras A, Hoffmann AA, Lemaitre B, et al 2015. Genetic, molecular and physiological basis of variation in Drosophila gut immunocompetence. Nat Commun. 6:7829. 10.1038/ncomms8829. 26213329 PMC4525169

[iyab005-B14] Branco J , Al-Ramahi I, Ukani L, Pérez AM, Fernandez-Funez P, et al 2008. Comparative analysis of genetic modifiers in Drosophila points to common and distinct mechanisms of pathogenesis among polyglutamine diseases. Hum Mol Genet. 17:376–390. 10.1093/hmg/ddm315. 17984172

[iyab005-B15] Calgaro S , Boube M, Cribbs DL, Bourbon H-M. 2002. The Drosophila gene taranis encodes a novel trithorax group member potentially linked to the cell cycle regulatory apparatus. Genetics. 160:547–560. 11861561 10.1093/genetics/160.2.547 PMC1461966

[iyab005-B16] Chapman JR , Dowell MA, Chan R, Unckless RL. 2020. The genetic basis of natural variation in *Drosophila melanogaster* immune defense against *Enterococcus faecalis*. Genes. 11:234. 10.3390/genes11020234. PMC7074548 32098395

[iyab005-B17] Chow CY , Kelsey KJP, Wolfner MF, Clark AG. 2016. Candidate genetic modifiers of retinitis pigmentosa identified by exploiting natural variation in *Drosophila*. Hum Mol Genet. 25:651–659. 10.1093/hmg/ddv502. 26662796 PMC4743685

[iyab005-B18] Chow CY , Wolfner MF, Clark AG. 2013. Using natural variation in Drosophila to discover previously unknown endoplasmic reticulum stress genes. Proc Natl Acad Sci U S A. 110:9013–9018. 10.1073/pnas.1307125110. 23667151 PMC3670321

[iyab005-B19] Coleman SA , Fischer ER, Howe D, Mead DJ, Heinzen RA. 2004. Temporal analysis of *Coxiella burnetii* morphological differentiation. J Bacteriol. 186:7344–7352. 10.1128/JB.186.21.7344-7352.2004. 15489446 PMC523218

[iyab005-B20] Cui Y , Ying Y, van Hasselt A, Ng KM, Yu J, et al 2008. OPCML is a broad tumor suppressor for multiple carcinomas and lymphomas with frequently epigenetic inactivation. PLoS One. 3:e2990. 10.1371/journal.pone.0002990. 18714356 PMC2500176

[iyab005-B21] Dahlgren FS , McQuiston JH, Massung RF, Anderson AD. 2015. Q fever in the United States: summary of case reports from two national surveillance systems, 2000-2012. Am J Trop Med Hyg. 92:247–255. 10.4269/ajtmh.14-0503. 25404080 PMC4347324

[iyab005-B22] De Lange MM , Schimmer B, Vellema P, Hautvast JL, Schneeberger PM, et al 2014. *Coxiella burnetii* seroprevalence and risk factors in sheep farmers and farm residents in The Netherlands. Epidemiol Infect. 142:1231–1244. 10.1017/s0950268813001726. 23920311 PMC4045170

[iyab005-B23] Delaby A , Gorvel L, Espinosa L, Lepolard C, Raoult D, et al 2012. Defective monocyte dynamics in Q fever granuloma deficiency. J Infect Dis. 205:1086–1094. 10.1093/infdis/jis013. 22351939

[iyab005-B24] Dolgachev V , Panicker S, Balijepalli S, McCandless LK, Yin Y, et al 2018. Electroporation-mediated delivery of FER gene enhances innate immune response and improves survival in a murine model of pneumonia. Gene Ther. 25:359–375. 10.1038/s41434-018-0022-y. 29907877 PMC6195832

[iyab005-B25] Dudzic JP , Hanson MA, Iatsenko I, Kondo S, Lemaitre B. 2019. More than black or white: melanization and toll share regulatory serine proteases in Drosophila. Cell Rep. 27:1050–1061.e3. 10.1016/j.celrep.2019.03.101. 31018123

[iyab005-B26] Duron O , Noel V, McCoy KD, Bonazzi M, Sidi-Boumedine K, et al 2015. The recent evolution of a maternally-inherited endosymbiont of ticks led to the emergence of the Q fever pathogen, *Coxiella burnetii*. PLoS Pathog. 11:e1004892. 10.1371/journal.ppat.1004892. 25978383 PMC4433120

[iyab005-B27] Everman ER , McNeil CL, Hackett JL, Bain CL, Macdonald SJ. 2019. Dissection of complex, fitness-related traits in multiple *Drosophila* mapping populations offers insight into the genetic control of stress resistance. Genetics. 211:1449–1467. 10.1534/genetics.119.301930. 30760490 PMC6456312

[iyab005-B28] Everman ER , Morgan TJ. 2018. Antagonistic pleiotropy and mutation accumulation contribute to age‐related decline in stress response. Evolution. 72:303–317. 10.1111/evo.13408. 29214647

[iyab005-B29] Fadista J , Manning AK, Florez JC, Groop L. 2016. The (in)famous GWAS P-value threshold revisited and updated for low-frequency variants. Eur J Hum Genet. 24:1202–1205. 10.1038/ejhg.2015.269. 26733288 PMC4970684

[iyab005-B30] Faugaret D , Ben Amara A, Alingrin J, Daumas A, Delaby A, et al 2014. Granulomatous response to *Coxiella burnetii*, the agent of Q fever: the lessons from gene expression analysis. Front Cell Infect Microbiol. 4:172. 10.3389/fcimb.2014.00172. 25566510 PMC4266094

[iyab005-B31] Fernandez-Funez P , Nino-Rosales ML, de Gouyon B, She W-C, Luchak JM, et al 2000. Identification of genes that modify ataxin-1-induced neurodegeneration. Nature. 408:101–106. 10.1038/35040584. 11081516

[iyab005-B32] Gelbart WM. 1989. The decapentaplegic gene: a TGF-beta homologue controlling pattern formation in Drosophila. Development. 107 Suppl:65–74. 2699859 10.1242/dev.107.Supplement.65

[iyab005-B33] Ghigo E , Capo C, Tung CH, Raoult D, Gorvel JP, et al 2002. *Coxiella burnetii* survival in THP-1 monocytes involves the impairment of phagosome maturation: IFN-gamma mediates its restoration and bacterial killing. J Immunol. 169:4488–4495. 12370385 10.4049/jimmunol.169.8.4488

[iyab005-B34] Gibson J , Russ TC, Clarke T-K, Howard DM, Hillary RF, et al 2019. A meta-analysis of genome-wide association studies of epigenetic age acceleration. PLoS Genet. 15:e1008104. 10.1371/journal.pgen.1008104. 31738745 PMC6886870

[iyab005-B35] Glennon NB , Jabado O, Lo MK, Shaw ML. 2015. Transcriptome profiling of the virus-induced innate immune response in *Pteropus vampyrus* and its attenuation by nipah virus interferon antagonist functions. J Virol. 89:7550–7566. 10.1128/JVI.00302-15. 25972557 PMC4505658

[iyab005-B36] Gorvel L , Textoris J, Banchereau R, Ben Amara A, Tantibhedhyangkul W, et al 2014. Intracellular bacteria interfere with dendritic cell functions: role of the type I interferon pathway. PLoS One. 9:e99420. 10.1371/journal.pone.0099420. 24915541 PMC4051653

[iyab005-B37] Goto A , Okado K, Martins N, Cai H, Barbier V, et al 2018. The kinase IKKβ regulates a STING- and NF-κB-dependent antiviral response pathway in Drosophila. Immunity. 49:225–234.e4. 10.1016/j.immuni.2018.07.013. 30119996 PMC6267954

[iyab005-B38] Graveley BR , Brooks AN, Carlson JW, Duff MO, Landolin JM, et al 2011. The developmental transcriptome of *Drosophila melanogaster*. Nature. 471:473–479. 10.1038/nature09715. 21179090 PMC3075879

[iyab005-B39] Grossmann A , Benlasfer N, Birth P, Hegele A, Wachsmuth F, et al 2015. Phospho‐tyrosine dependent protein–protein interaction network. Mol Syst Biol. 11:794. 10.15252/msb.20145968. 25814554 PMC4380928

[iyab005-B40] He BZ , Ludwig MZ, Dickerson DA, Barse L, Arun B, et al 2014. Effect of genetic variation in a *Drosophila* model of diabetes-associated misfolded human proinsulin. Genetics. 196:557–567. 10.1534/genetics.113.157800. 24281155 PMC3914626

[iyab005-B41] Hedges JF , Robison A, Kimmel E, Christensen K, Lucas E, et al 2016. Type I interferon counters or promotes *Coxiella burnetii* replication dependent on tissue. Infect Immun. 84:1815–1825. 10.1128/IAI.01540-15. 27068091 PMC4907146

[iyab005-B42] Hein MY , Hubner NC, Poser I, Cox J, Nagaraj N, et al 2015. A human interactome in three quantitative dimensions organized by stoichiometries and abundances. Cell. 163:712–723. 10.1016/j.cell.2015.09.053. 26496610

[iyab005-B43] Hiroyasu A , DeWitt DC, Goodman AG. 2018. Extraction of hemocytes from *Drosophila melanogaster* larvae for microbial infection and analysis. J Vis Exp. 135:57077. 10.3791/57077. PMC6101378 29889203

[iyab005-B44] Hoffmann JA. 2003. The immune response of Drosophila. Nature. 426:33–38. 10.1038/nature02021. 14603309

[iyab005-B45] Howick VM , Lazzaro BP. 2017. The genetic architecture of defence as resistance to and tolerance of bacterial infection in *Drosophila melanogaster*. Mol Ecol. 26:1533–1546. 10.1111/mec.14017. 28099780

[iyab005-B46] Hu Y , Flockhart I, Vinayagam A, Bergwitz C, Berger B, et al 2011. An integrative approach to ortholog prediction for disease-focused and other functional studies. BMC Bioinformatics. 12:357. 10.1186/1471-2105-12-357. 21880147 PMC3179972

[iyab005-B6936751] Hua X, Li B, Song L, Hu C, Li X , et al 2018. Stimulator of interferon genes (STING) provides insect antiviral immunity by promoting Dredd caspase–mediated NF-κB activation. Journal of Biological Chemistry. 293:11878–11890. 10.1074/jbc.RA117.000194 PMC6066306 29875158

[iyab005-B47] Huang W , Massouras A, Inoue Y, Peiffer J, Ramia M, et al 2014. Natural variation in genome architecture among 205 *Drosophila melanogaster* genetic reference panel lines. Genome Res. 24:1193–1208. 10.1101/gr.171546.113. 24714809 PMC4079974

[iyab005-B48] Jeacock L , Faria J, Horn D. 2018. Codon usage bias controls mRNA and protein abundance in trypanosomatids. eLife. 7:e32496. 10.7554/eLife.32496. PMC5896881 29543155

[iyab005-B49] Ka MB , Gondois-Rey F, Capo C, Textoris J, Million M, et al 2014. Imbalance of circulating monocyte subsets and PD-1 dysregulation in Q fever endocarditis: the role of IL-10 in PD-1 modulation. PLoS One. 9:e107533. 10.1371/journal.pone.0107533. 25211350 PMC4161472

[iyab005-B50] Karakousis PC , Trucksis M, Dumler JS. 2006. Chronic Q fever in the United States. J Clin Microbiol. 44:2283–2287. 10.1128/jcm.02365-05. 16757641 PMC1489455

[iyab005-B51] Kelsey KJP , Clark AG. 2017. Variation in position effect variegation within a natural population. Genetics. 207:1157–1166. 10.1534/genetics.117.300306. 28931559 PMC5676239

[iyab005-B52] Kersh GJ , Fitzpatrick KA, Self JS, Priestley RA, Kelly AJ, et al 2013. Presence and persistence of *Coxiella burnetii* in the environments of goat farms associated with a Q fever outbreak. Appl Environ Microbiol. 79:1697–1703. 10.1128/aem.03472-12. 23315737 PMC3591968

[iyab005-B53] Komar AA. 2016. The Yin and Yang of codon usage. Hum Mol Genet. 25:R77–R85. 10.1093/hmg/ddw207. 27354349 PMC6372012

[iyab005-B54] Královičová J , Houngninou-Molango S, Krämer A, Vořechovský I. 2004. Branch site haplotypes that control alternative splicing. Hum Mol Genet. 13:3189–3202. 10.1093/hmg/ddh334. 15496424

[iyab005-B55] Lavoy S , Chittoor-Vinod VG, Chow CY, Martin I. 2018. Genetic modifiers of neurodegeneration in a *Drosophila* model of Parkinson’s disease. Genetics. 209:1345–1356. 10.1534/genetics.118.301119. 29907646 PMC6063243

[iyab005-B56] Lemaitre B , Hoffmann J. 2007. The host defense of *Drosophila melanogaster*. Annu Rev Immunol. 25:697–743. 10.1146/annurev.immunol.25.022106.141615. 17201680

[iyab005-B57] Leone M , Honstettre A, Lepidi H, Capo C, Bayard F, et al 2004. Effect of sex on *Coxiella burnetii* infection: protective role of 17β-estradiol. J Infect Dis. 189:339–345. 10.1086/380798. 14722900

[iyab005-B58] Li P , Ma Z, Yu Y, Hu X, Zhou Y, et al 2019. FER promotes cell migration via regulating JNK activity. Cell Prolif. 52:10.1111/cpr.12656. PMC6797522 31264309

[iyab005-B59] Mackay TF , Richards S, Stone EA, Barbadilla A, Ayroles JF, et al 2012. The *Drosophila melanogaster* genetic reference panel. Nature. 482:173–178. 10.1038/nature10811. 22318601 PMC3683990

[iyab005-B60] Mackay TFC , Huang W. 2018. Charting the genotype-phenotype map: lessons from the *Drosophila melanogaster* genetic reference panel: charting the genotype-phenotype map. WIREs Dev Biol. 7:e289. 10.1002/wdev.289. PMC5746472 28834395

[iyab005-B61] Madariaga MG , Rezai K, Trenholme GM, Weinstein RA. 2003. Q fever: a biological weapon in your backyard. Lancet Infect Dis. 3:709–721. 14592601 10.1016/s1473-3099(03)00804-1

[iyab005-B62] Magwire MM , Fabian DK, Schweyen H, Cao C, Longdon B, et al 2012. Genome-wide association studies reveal a simple genetic basis of resistance to naturally coevolving viruses in *Drosophila melanogaster*. PLoS Genet. 8:e1003057. 10.1371/journal.pgen.1003057. 23166512 PMC3499358

[iyab005-B63] Marrie TJ , Stein A, Janigan D, Raoult D. 1996. Route of infection determines the clinical manifestations of acute Q fever. J Infect Dis. 173:484–487. 8568318 10.1093/infdis/173.2.484

[iyab005-B64] Martin M , Hiroyasu A, Guzman RM, Roberts SA, Goodman AG. 2018. Analysis of Drosophila STING reveals an evolutionarily conserved antimicrobial function. Cell Rep. 23:3537–3550.e6. 10.1016/j.celrep.2018.05.029. 29924997 PMC6114933

[iyab005-B65] Martins NE , Faria VG, Teixeira L, Magalhães S, Sucena É. 2013. Host adaptation is contingent upon the infection route taken by pathogens. PLoS Pathog. 9:e1003601. 10.1371/journal.ppat.1003601. 24086131 PMC3784483

[iyab005-B66] Maurin M , Raoult D. 1999. Q fever. Clin Microbiol Rev. 12:518–553. 10515901 10.1128/cmr.12.4.518 PMC88923

[iyab005-B67] McCracken AW , Buckle E, Simons MJP. 2020. The relationship between longevity and diet is genotype dependent and sensitive to desiccation in *Drosophila melanogaster*. J Exp Biol. 223:jeb230185. doi:10.1242/jeb.230185. 10.1242/jeb.230185 PMC7725603 33109715

[iyab005-B68] McQuiston JH , Childs JE, Thompson HA. 2002. Q fever. J Am Veter Med Assoc. 221:796–799. 10.2460/javma.2002.221.796 12322916

[iyab005-B69] Meghari S , Bechah Y, Capo C, Lepidi H, Raoult D, et al 2008. Persistent *Coxiella burnetii* infection in mice overexpressing IL-10: an efficient model for chronic Q fever pathogenesis. PLoS Pathog. 4:e23. 10.1371/journal.ppat.0040023. 18248094 PMC2222951

[iyab005-B70] Mehraj V , Textoris J, Ben Amara A, Ghigo E, Raoult D, et al 2013. Monocyte responses in the context of Q fever: from a static polarized model to a kinetic model of activation. J Infect Dis. 208:942–951. 10.1093/infdis/jit266. 23801603

[iyab005-B71] Miles LA , Baik N, Lighvani S, Khaldoyanidi S, Varki NM, et al 2017. Deficiency of plasminogen receptor, Plg-R _KT_, causes defects in plasminogen binding and inflammatory macrophage recruitment *in vivo*. J Thromb Haemost. 15:155–162. 10.1111/jth.13532. 27714956 PMC5280214

[iyab005-B72] Millar JA , Valdés R, Kacharia FR, Landfear SM, Cambronne ED, et al 2015. *Coxiella burnetii* and *Leishmania mexicana* residing within similar parasitophorous vacuoles elicit disparate host responses. Front Microbiol. 6:10.3389/fmicb.2015.00794. PMC4528172 26300862

[iyab005-B73] Murray MJ. 2006. The Fes/Fer non-receptor tyrosine kinase cooperates with Src42A to regulate dorsal closure in Drosophila. Development. 133:3063–3073. 10.1242/dev.02467. 16831834

[iyab005-B74] Nazario-Toole AE. 2016. Genome wide association studies of phagocytosis and the cellular immune response in *Drosophila melanogaster*. Digital Repository at the University of Maryland. 10.13016/M26V1H.

[iyab005-B75] Omsland A , Cockrell DC, Howe D, Fischer ER, Virtaneva K, et al 2009. Host cell-free growth of the Q fever bacterium *Coxiella burnetii*. Proc Natl Acad Sci U S A. 106:4430–4434. 10.1073/pnas.0812074106. 19246385 PMC2657411

[iyab005-B76] Palu RAS , Chow CY. 2018. Baldspot/ELOVL6 is a conserved modifier of disease and the ER stress response. PLoS Genet. 14:e1007557. 10.1371/journal.pgen.1007557. 30081392 PMC6078684

[iyab005-B77] Palu RAS , Dalton HM, Chow CY. 2020. Decoupling of apoptosis from activation of the ER stress response by the *Drosophila* Metallopeptidase superdeath. Genetics. 214:913–925. 10.1534/genetics.119.303004. 32047096 PMC7153941

[iyab005-B78] Palu RAS , Ong E, Stevens K, Chung S, Owings KG, et al 2019. Natural genetic variation screen in *Drosophila* identifies wnt signaling, mitochondrial metabolism, and redox homeostasis genes as modifiers of. Apoptosis. G3 (Bethesda). 9:3995–4005. 10.1534/g3.119.400722. 31570502 PMC6893197

[iyab005-B79] Pennings JLA , Kremers MNT, Hodemaekers HM, Hagenaars JCJP, Koning OHJ, et al 2015. Dysregulation of serum gamma interferon levels in vascular chronic Q Fever patients provides insights into disease pathogenesis. Clin Vaccine Immunol. 22:664–671. 10.1128/CVI.00078-15. 25924761 PMC4446408

[iyab005-B80] Pinheiro J , Bates D, DebRoy S, Sarkar D, R Core Team. 2020. nlme: linear and nonlinear mixed effects models. R Package Version. 3.1-151. http://CRAN.R-project.org/package=nlme.

[iyab005-B81] Pitchers W , Nye J, Márquez EJ, Kowalski A, Dworkin I, et al 2019. A multivariate genome-wide association study of wing shape in *Drosophila melanogaster*. Genetics. 211:1429–1447. 10.1534/genetics.118.301342. 30792267 PMC6456314

[iyab005-B82] Raoult D , Marrie T, Mege J. 2005. Natural history and pathophysiology of Q fever. Lancet Infect Dis. 5:219–226. 10.1016/s1473-3099(05)70052-9. 15792739

[iyab005-B83] Regan JC , Brandão AS, Leitão AB, Mantas Dias ÂR, Sucena É, et al 2013. Steroid hormone signaling is essential to regulate innate immune cells and fight bacterial infection in Drosophila. PLoS Pathog. 9:e1003720. 10.1371/journal.ppat.1003720. 24204269 PMC3812043

[iyab005-B84] Roest HI , Bossers A, van Zijderveld FG, Rebel JM. 2013. Clinical microbiology of *Coxiella burnetii* and relevant aspects for the diagnosis and control of the zoonotic disease Q fever. Vet Q. 33:148–160. 10.1080/01652176.2013.843809. 24161079

[iyab005-B85] Roest HI , Ruuls RC, Tilburg JJ, Nabuurs-Franssen MH, Klaassen CH, et al 2011a. Molecular epidemiology of *Coxiella burnetii* from ruminants in Q fever outbreak, the Netherlands. Emerg Infect Dis. 17:668–675. 10.3201/eid1704.101562. 21470457 PMC3377418

[iyab005-B86] Roest HI , Tilburg JJ, van der Hoek W, Vellema P, van Zijderveld FG, et al 2011b. The Q fever epidemic in The Netherlands: history, onset, response and reflection. Epidemiol Infect. 139:1–12. 10.1017/s0950268810002268. 20920383

[iyab005-B87] Roest HJ , van Gelderen B, Dinkla A, Frangoulidis D, van Zijderveld F, et al 2012. Q fever in pregnant goats: pathogenesis and excretion of *Coxiella burnetii*. PLoS One. 7:e48949. 10.1371/journal.pone.0048949. 23152826 PMC3494687

[iyab005-B88] Salinas RP , Ortiz Flores RM, Distel JS, Aguilera MO, Colombo MI, et al 2015. *Coxiella burnetii* phagocytosis is regulated by GTPases of the Rho family and the RhoA effectors mDia1 and ROCK. PLoS One. 10:e0145211–e0145211. 10.1371/journal.pone.0145211. 26674774 PMC4682630

[iyab005-B89] Sauna ZE , Kimchi-Sarfaty C. 2011. Understanding the contribution of synonymous mutations to human disease. Nat Rev Genet. 12:683–691. 10.1038/nrg3051. 21878961

[iyab005-B90] Savitz J , Frank MB, Victor T, Bebak M, Marino JH, et al 2013. Inflammation and neurological disease-related genes are differentially expressed in depressed patients with mood disorders and correlate with morphometric and functional imaging abnormalities. Brain Behav Immun. 31:161–171. 10.1016/j.bbi.2012.10.007. 23064081 PMC3577998

[iyab005-B91] Schimmer B , Morroy G, Dijkstra F, Schneeberger PM, Weers-Pothoff G, et al 2008. Large Ongoing Q Fever Outbreak in the South of The Netherlands, 2008. Euro surveillance: bulletin Europeen sur les maladies transmissibles = European communicable disease bulletin 13. 18761906

[iyab005-B92] Schmidt JM , Battlay P, Gledhill-Smith RS, Good RT, Lumb C, et al 2017. Insights into DDT resistance from the *Drosophila melanogaster* genetic reference panel. Genetics. 207:1181–1193. 10.1534/genetics.117.300310. 28935691 PMC5676240

[iyab005-B93] Schoffelen T , Ammerdorffer A, Hagenaars JC, Bleeker-Rovers CP, Wegdam-Blans MC, et al 2015. Genetic variation in pattern recognition receptors and adaptor proteins associated with development of chronic Q fever. J Infect Dis. 212:818–829. 10.1093/infdis/jiv113. 25722298

[iyab005-B94] Sheehan G , Garvey A, Croke M, Kavanagh K. 2018. Innate humoral immune defences in mammals and insects: The same, with differences? Virulence. 9:1625–1639. 10.1080/21505594.2018.1526531. 30257608 PMC7000196

[iyab005-B95] Singh RK , Cooper TA. 2012. Pre-mRNA splicing in disease and therapeutics. Trends Mol Med. 18:472–482. 10.1016/j.molmed.2012.06.006. 22819011 PMC3411911

[iyab005-B96] Sondgeroth KS , Davis MA, Schlee SL, Allen AJ, Evermann JF, et al 2013. Seroprevalence of *Coxiella burnetii* in Washington State domestic goat herds. Vector Borne Zoonotic Dis. 13:779–783. 10.1089/vbz.2013.1331. 24107207 PMC5695728

[iyab005-B97] Stranger BE , Stahl EA, Raj T. 2011. Progress and promise of genome-wide association studies for human complex trait genetics. Genetics. 187:367–383. 10.1534/genetics.110.120907. 21115973 PMC3030483

[iyab005-B98] Tafesh-Edwards G , Eleftherianos I. 2020. Drosophila immunity against natural and nonnatural viral pathogens. Virology. 540:165–171. 10.1016/j.virol.2019.12.001. 31928998

[iyab005-B99] Talsness DM , Owings KG, Coelho E, Mercenne G, Pleinis JM, et al 2020. A Drosophila screen identifies NKCC1 as a modifier of NGLY1 deficiency. eLife. 9:e57831. 10.7554/eLife.57831. PMC7758059 33315011

[iyab005-B100] Textoris J , Ban LH, Capo C, Raoult D, Leone M, et al 2010. Sex-related differences in gene expression following *Coxiella burnetii* infection in mice: potential role of circadian rhythm. PLoS One. 5:e12190. 10.1371/journal.pone.0012190. 20730052 PMC2921390

[iyab005-B101] Vago JP , Sugimoto MA, Lima KM, Negreiros-Lima GL, Baik N, et al 2019. Plasminogen and the plasminogen receptor, Plg-RKT, regulate macrophage phenotypic, and functional changes. Front Immunol. 10:1458. 10.3389/fimmu.2019.01458. 31316511 PMC6611080

[iyab005-B102] van Asseldonk MA , Prins J, Bergevoet RH. 2013. Economic assessment of Q fever in the Netherlands. Prev Vet Med. 112:27–34. 10.1016/j.prevetmed.2013.06.002. 23866818

[iyab005-B103] Wang JB , Lu H-L, St Leger RJ. 2017. The genetic basis for variation in resistance to infection in the *Drosophila melanogaster* genetic reference panel. PLoS Pathog. 13:e1006260. 10.1371/journal.ppat.1006260. 28257468 PMC5352145

[iyab005-B104] Wang Z , Burge CB. 2008. Splicing regulation: From a parts list of regulatory elements to an integrated splicing code. RNA. 14:802–813. 10.1261/rna.876308. 18369186 PMC2327353

[iyab005-B105] Weber MM , Faris R, van Schaik EJ, McLachlan JT, Wright WU, et al 2016. The type IV secretion system effector protein CirA Stimulates the GTPase activity of RhoA and is required for virulence in a mouse model of *Coxiella burnetii* infection. Infect Immun. 84:2524–2533. 10.1128/IAI.01554-15. 27324482 PMC4995899

[iyab005-B106] Wielders CC , Hackert VH, Schimmer B, Hodemaekers HM, de Klerk A, et al 2015. Single nucleotide polymorphisms in immune response genes in acute Q fever cases with differences in self-reported symptoms. Eur J Clin Microbiol Infect Dis. 34:943–950. 10.1007/s10096-014-2310-9. 25577174 PMC7088184

[iyab005-B107] Will CL , Luhrmann R. 2011. Spliceosome structure and function. Cold Spring Harbor Perspect Biol. 3:a003707. 10.1101/cshperspect.a003707. PMC3119917 21441581

[iyab005-B108] Yano T , Mita S, Ohmori H, Oshima Y, Fujimoto Y, et al 2008. Autophagic control of listeria through intracellular innate immune recognition in Drosophila. Nat Immunol. 9:908–916. 10.1038/ni.1634. 18604211 PMC2562576

[iyab005-B109] Yoo S , Nair S, Kim H, Kim Y, Lee C, et al 2020. Knock-in mutations of scarecrow, a Drosophila homolog of mammalian Nkx2.1, reveal a novel function required for development of the optic lobe in *Drosophila melanogaster*. Dev Biol. 461:145–159. 10.1016/j.ydbio.2020.02.008. 32061586

[iyab005-B110] Zhang Y-B , Hu J, Zhang J, Zhou X, Li X, et al 2016. Genome-wide association study identifies multiple susceptibility loci for craniofacial microsomia. Nat Commun. 7:10605. 10.1038/ncomms10605. 26853712 PMC4748111

[iyab005-B111] Zhou Z , Dang Y, Zhou M, Li L, Yu C, et al 2016. Codon usage is an important determinant of gene expression levels largely through its effects on transcription. Proc Natl Acad Sci U S A. 113:E6117–E6125. 10.1073/pnas.1606724113. 27671647 PMC5068308

